# Recent Studies on Ponatinib in Cancers Other Than Chronic Myeloid Leukemia

**DOI:** 10.3390/cancers10110430

**Published:** 2018-11-09

**Authors:** Francesca Musumeci, Chiara Greco, Giancarlo Grossi, Alessio Molinari, Silvia Schenone

**Affiliations:** 1 Department of Pharmacy, University of Genova, Viale Benedetto XV 3, 16132 Genova, Italy; francesca.musumeci@unige.it (F.M.); greco.phd@difar.unige.it (C.G.); grossig@unige.it (G.G.); 2 Department of Biotechnology, Chemistry and Pharmacy, University of Siena, Via Aldo Moro 2, 53100 Siena, Italy; molinari.a2@gmail.com

**Keywords:** ponatinib, protein kinase inhibitor, cancer

## Abstract

Ponatinib is a third line drug for the treatment of chronic myeloid leukemia patients, especially those that develop the gatekeeper mutation T315I, which is resistant to the first and the second line drugs imatinib, nilotinib, dasatinib and bosutinib. The compound was first identified as a pan Bcr-Abl and Src kinase inhibitor. Further studies have indicated that it is a multitargeted inhibitor that is active on FGFRs, RET, AKT, ERK1/2, KIT, MEKK2 and other kinases. For this reason, the compound has been evaluated on several cancers in which these kinases play important roles, including thyroid, breast, ovary and lung cancer, neuroblastoma, rhabdoid tumours and in myeloproliferative disorders. Ponatinib is also being tested in clinical trials to evaluate its activity in FLT3-ITD acute myelogenous leukemia, head and neck cancers, certain type of lung cancer, gastrointestinal stromal tumours and other malignancies. In this review we report the most recent preclinical and clinical studies on ponatinib in cancers other than CML, with the aim of giving a complete overview of this interesting compound.

## 1. Introduction

**Figure 1:**
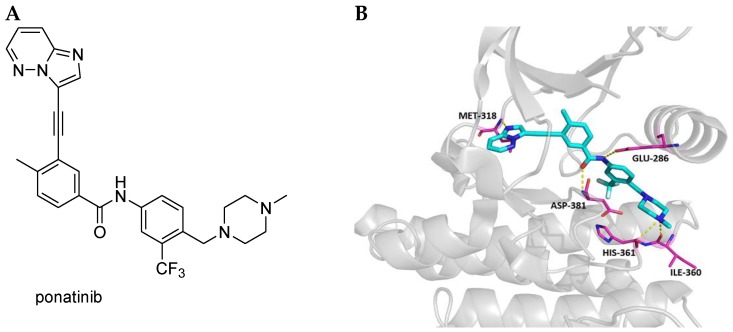
(**A**) Chemical structure of ponatinib. (**B**) Binding mode of ponatinib with Bcr-Abl. Hydrogen bonds are represented as yellow dashed lines.

Ponatinib, AP24534 (Figure 1) was synthesized by Ariad Pharmaceuticals Inc., Cambridge, MA, USA [1]. This imidazo[1,2-*b*]pyridazine has been initially identified as a nanomolar inhibitor of the constitutively activated cytoplasmic tyrosine kinase (TK) Bcr-Abl, which is codified by the chromosome Philadelphia (Ph). Bcr-Abl is present in 90% of chronic myeloid leukemia (CML) and in 10% of Ph^+^ acute lymphoblastic leukemia (ALL) patients [2] and it is a well-known target to treat these diseases. Ponatinib is a third generation Bcr-Abl inhibitor, which is active on native Abl with an IC_50_ value of 0.37 nM and the mutant T315I with an IC_50_ value of 2.0 nM [3]. Its activity is particularly important, since no other drug is able to inhibit this mutant, which very frequently arises during the treatment with the other TK inhibitors (imatinib, dasatinib, nilotinib and bosutinib) approved for CML and/or Ph^+^ ALL treatment and confers drug resistance. In particular, the T315I mutation occurs more frequently in Ph^+^ ALL patients than in CML patients [4]. Ponatinib is also active against other clinically important Bcr-Abl mutants with IC_50_ values in the range 0.30–0.44 nM [1]. The drug is prescribed at a dose of 45 mg daily, per os and its use is continued as long as there is no evidence of disease progression or unacceptable toxicity. It is not recommended for the treatment of patients with newly diagnosed chronic phase CML. The dose is reduced to 30 mg daily in patients with hepatic impairment. Caution is recommended when ponatinib is co-administered with strong CYP3A inhibitors, such as macrolide antibiotics and azolic antimycotics and in patients with poor renal function. It cannot be used during pregnancy or breastfeeding [5].

Ponatinib activity on T315I Bcr-Abl is probably due to the presence of the inflexible acetylene linkage, which is peculiar to this molecule. This rigid structure reduces steric repulsions with the bulk of the isoleucine side chain of the T315I mutant and is involved in favourable van der Waals interactions with I315. Ponatinib is a typical type II inhibitor, in fact, it targets the catalytic site and an adjacent allosteric site available only in the inactive enzymatic form [6]. Specifically, the imidazo[1,2-*b*]pyridazine core occupies the adenine pocket of the enzyme, the methylphenyl group fits into the hydrophobic pocket behind the gatekeeper residue and the trifluoromethylphenyl substituent tightly binds the pocket induced by the DFG-out, inactive, conformation of the protein. Ariad researchers determined the crystal structure of ponatinib bound to Bcr-Abl [7]. An image of this structure (from Protein Data Bank, PDB 3OXZ) is reported in Figure 1.

In preclinical trials and adsorption, distribution, metabolism and excretion (ADME) studies, it was evidenced that the piperazine tail confers high potency and good aqueous solubility with reduced plasma protein binding, leading to good pharmacokinetic properties following oral dosing in rats and mice [1]. Daily oral administration of the compound significantly prolongs survival of mice intravenously injected with Bcr-Abl T315I-expressing Ba/F3 cells [1].

In 2012, ponatinib has been approved (commercial name Iclusig^®^) by the U.S. Food and Drug Administration (FDA) for the treatment of resistant or imatinib intolerant CML and Ph+ ALL patients, especially those harbouring the T315I mutation but the drug was soon temporarily suspended because of the increasing numbers of vascular events observed in ponatinib-treated patients [8].

In December 2013 the drug was reauthorized by the FDA after a revised indication statement and a boxed warming, with alerts to the risk of vascular occlusive events and heart failure [9]. In July 2013, it was also approved by the EMA [10].

Ponatinib is not selective for Abl but is a multikinase inhibitor, with high affinity to 40 kinases. Indeed, it inhibits vascular endothelial growth factor receptor (VEGFR), platelet-derived growth factor receptor (PDGFR), fibroblast growth factor receptor (FGFR), Src, KIT, RET, AKT, ERK, FLT3, EphR, MEKK2 and other kinases with IC_50_ values in the range 0.1 and 20 nM. On the other hand, it does not inhibit Aurora kinase family members, nor insulin receptor or cyclin-dependent kinase 2 [1].

Until now, X-ray structures of ponatinib bound with different kinases (FGFR1, PDB 4V04; FGFR4, PDB 4UXQ; KIT, PDB 4U0I) have been obtained and confirm that the compound is a type II inhibitor. For many other kinases targeted by it, including RET, the ponatinib-bound X-ray structures are not yet available [11]. 

Ponatinib has been evaluated for the treatment of different diseases, usually malignancies, where one of more of the cited kinases play important roles, including thyroid, breast, ovary and lung cancer, neuroblastoma (NB), rhabdoid tumours and in myeloproliferative disorders. Moreover, ponatinib is being tested in clinical trials to evaluate its activity in FLT3-internal tandem duplication (ITD) acute myelogenous leukemia, head and neck cancers, certain type of lung cancer, gastrointestinal stromal tumours and other malignancies.

Many recent and interesting reviews summarize the activity of ponatinib in CML and Ph^+^ ALL [12,13,14,15] and for this reason we do not treat this topic.

In this review we report preclinical and clinical studies on ponatinib in cancers other than CML, with the aim of giving a complete overview of this interesting compound. To write this article we performed a combined research on Scifinder and PubMed and reported the main articles from 2012. We classified the studies on the basis of the malignancy on which ponatinib has been tested, in correlation with the kinase(s) inhibited by the drug.

Table 1 summarizes the cancers on which the drug has been evaluated and the targeted kinase(s).

A separate section is devoted to the clinical trials active at the moment of writing.

## 2. Haematological Tumours

### 2.1. Acute Myeloid Leukemia

Acute myeloid leukemia (AML) is frequently characterized by mutations of the receptor TK FLT3. In particular ITD mutations in FLT3 juxtamembrane domain are correlated with poor prognosis and are present in a high percentage of patients [16].

Preclinical studies demonstrated that ponatinib is active against FLT3-mutated cell lines of AML and in the MV4-11 xenograft mouse model [17].

Several point mutations in the FLT3 tyrosine kinase domain (TKD) conferring resistance to different FLT3 inhibitors have been successively identified. Ponatinib induces apoptosis not only in the parental FLT3-ITD cell line but also in cells harbouring additional FLT3TKD point mutations, including N676D, F691I or G697R, with IC_50_ values in the range 4–77 nM. Consistently, the compound inhibits FLT3 downstream proteins, including STAT5, AKT and ERK1/2 [18]. 

Smith et al. further confirmed that ponatinib possesses in vitro activity against the clinically relevant FLT3-ITD/F691L, which is the most commonly detected FLT3-ITD mutation and is involved in in vitro resistance to other FLT3 inhibitors currently in clinical development. On the other hand, mutations in the D835 position have been found to confer resistance to ponatinib [19]. More recently, Dovey et al. identified the FLT3 mutation F692L in a mouse model. This mutation corresponds to the human FLT3 F691L gatekeeper mutation just reported. The authors found that the growth of primary AML cell from mice bearing the F692L mutant is inhibited by ponatinib but not by other TK inhibitors such as sorafenib and quizartinib [20].

Encouraging results from a phase I clinical trial performed in 2013 indicated ponatinib as a potential drug for the treatment of patients with refractory AML [20]. Furthermore, a more recent study evaluated the use of ponatinib in four FLT3-ITD positive ALM patients after the failure of sorafenib. Unfortunately, the occurrence of resistant mutations, such as D835, are a common mechanism of resistance for both TK inhibitors. No hematologic response, bone marrow response nor remission were observed after ponatinib treatment. The toxicity during ponatinib treatment was reported as grade III/IV, based on common toxicity criteria, with a case of pancreatitis [21]. However, this study was conducted on a very small number of patients. Front line studies with ponatinib show clinical efficacy comparable to other non-selective FLT3 inhibitors such as midostaurin and sorafenib.

### 2.2. T-Cell Acute Lymphoblastic Leukemia

Recently, it has been reported that ponatinib is active in in vitro and in vivo models of T-cell acute lymphoblastic leukemia (T-ALL), when used in combination with PIM inhibitors [21]. PIM is a serine-threonine kinase [22] overexpressed in the majority of early T-cell precursor. The two PIM inhibitors namely AZD1208 [23] and LGB321 [24] have a potent antiproliferative effect on different T-ALL cell lines but they increase ERK and STAT5 phosphorylation, as a result of PIM inhibition and this is an unfavourable event during the research of anticancer therapies. As previously reported [18,25], ponatinib, by inhibiting kinases upstream of ERK and STAT5, reduces the phosphorylation of these two proteins. For this reason the association of ponatinib with PIM inhibitors possesses a synergistic antiproliferative and apoptotic activity in ALL cells. When tested in mice, the combination decreases the tumour mass and improves animal survival [21].

### 2.3. Chronic Eosinophilic Leukemia

A subset of chronic eosinophilic leukemia (CEL) patients bears mutated and/or fused forms of PDGFRα, which is a receptor TK targeted by ponatinib.

Lierman et al. found that the drug has antiproliferative activity on Ba/F3 cells expressing the imatinib-resistant FIP1L1-PDGFRαT674I or the pan-resistant FIP1L1-PDGFRα-D842V, which have been detected in a number of patients affected by CEL. Western blotting demonstrated that the mechanism of action of the compound is the inhibition of the phosphorylation of these mutated kinases. Importantly, ponatinib resulted inactive towards the WT Ba/F3 cells [26].

Jin et al. confirmed the activity of ponatinib on T674I FIP1L1-PDGFRα and the PDGFRα downstream substrates, including STAT3 and STAT5 [27]. The drug has antiproliferative effects on WT and T674I FIP1L1-PDGFRα-carrying CEL cells, with IC_50_ values in the range 0.004–2.5 nM. Moreover ponatinib shows proapoptotic effects in these cells and inhibits the phosphorylation and the level of β-catenin. The authors underlined the fact that the anticancer activity of ponatinib can be due, at least in part, to its effect on β-catenin. In vivo, ponatinib reduces the growth of xenografted BaF3-T674I FIP1L1-PDGFRα cells implanted in nude mice.

Similar results have been obtained by Sadovnik et al., who demonstrated that ponatinib is a potent antiproliferative agent of neoplastic eosinophils (EOL-1 cells) carrying the fusion protein FIP1L1-PDGFRα, which has been detected in CEL patients [28]. The compound shows IC_50_ values in the range 0.1–0.2 nM on EOL-1 cells and acts by inhibiting PDGFRα and PDGFR-downstream pathways. Moreover it induces apoptosis, reduces cell migration and results to be the most active compound among other PDGFR inhibitors. Ponatinib resulted also active on Ba/F3 cells expressing the two clinically relevant mutant forms of FIP1L1-PDGFRα, T674I and D842V, as previously reported by Lierman [26].

A limited number of CEL patients harbouring the fusion protein FIP1L1-PDGFα and its mutants also expresses high level of the protein hairy enhancer of split 1 (Hes1), which influences cell proliferation. Importantly, ponatinib possesses antiproliferative effects on cells harbouring both Hes1 and imatinib-resistant FLP1L1-PDGFRα mutants in vitro and in vivo [29].

### 2.4. Myeloproliferative Syndrome

The 8p11 myeloproliferative syndrome is a rare, aggressive neoplasm associated with eosinophilia and T or B lymphoblastic leukemia or lymphoma. This pathology is characterized by fused forms of FGFR1.

Ponatinib is a pan-FGFR inhibitor. The transmembrane TK FGFR family is constituted by four members (FGFR-1, -2, -3, -4). These enzymes, especially in their mutated or fused variants, have been implicated in many cancers [30,31]. Tucker et al. studied the binding mode of ponatinib with FGFR-1 and FGFR-4 and found that it binds with the DFG-out conformation of the enzymes, in a similar fashion of its binding mode with Bcr-Abl [32]. So, ponatinib, differently from the other available FGFR inhibitors, is a type II inhibitor.

The FGFR1 fusion proteins more frequently present in certain type of leukemia and lymphomas correlated with the 8p11 myeloproliferative syndrome are ZMYM2-FGFR1, Bcr-FGFR1, CEP110-FGFR1, FGFR1OP2-FGFR1, FOP-FGFR1 and CUX1-FGFR1, which are constitutively activated. At the moment 14 different FGFR1-partner genes have been described [33]. Ponatinib, at doses lower than 50 nM, inhibits the phosphorylation of these fusion proteins and of their downstream proteins (PLCγ, STAT5, Src) in cells, including transformed murine BaF3 cells, human KG1 cells and newly established murine SCLL (small cell lymphocytic lymphoma) cell lines [34]. In these cells the compound induces cell cycle arrest and apoptosis, while it is inactive on the corresponding wild type (WT) cells. The compound is also active in SCLL and KG1 murine models. In particular, ponatinib significantly delays or prevents tumorigenesis of KG1 cells in xenotransplanted mice, at a dose of 30 mg/kg.

In a similar study, Chase et al. investigated the antiproliferative activity of ponatinib on Ba/F3 cells transformed by ZMYM2-FGFR1 and Bcr-FGFR1 and on the FGFR1OP2-FGFR1 positive KG1A cell line. All the cell lines treated with ponatinib show reduced proliferation and decreased survival compared to the control. Ponatinib induces apoptosis and reduces phosphorylation of the FGFR1 fusion proteins and their substrates. Similar positive results have been obtained on cells derived from 8p11 myeloproliferative syndrome patients [35].

Among FGFR1 fused forms, TPR (translocated promoter region)-FGFR1 fusion is a rare but recurrent event in 8p11 myeloproliferative syndrome. The TPR-FGFR1 fusion protein leads to cytokine-independent cell survival, proliferation and granulocytic differentiation, which are events occurring in the chronic phase of myeloproliferative syndrome, that are characterized by myeloid hyperplasia. Malli et al. investigated the transforming potential and localization of the chimeric TPR-FGFR1 kinase and evaluated the activity of ponatinib and infigratinib (a potent and specific pan-FGFR inhibitor currently in clinical trials [36]) on the survival and proliferation of myeloid progenitor 32Dcl3 cells transformed with the TPR-FGFR1 fusion protein. Both compounds show antiproliferative effects on 32Dcl3 cells, with IC_50_ values of 49.8 and 7.7 nM, respectively while are inactive on normal cells [37]. From the just reported studies, ponatinib results as a promising compound for the treatment of this neoplasm.

Ponatinib was also tested in a patient with a FGFR1-rearranged neoplasm manifesting as a Bcr-FGFR1-positive trilineage T/B/myeloid mixed-phenotype acute leukemia [38]. The compound was administered at a dose of 45 mg daily, as a single agent, on an off-label basis, given the severity of the disease. The compound led to a substantial reduction of lymphadenopathy. However ponatinib, even if combined with conventional chemotherapy, was insufficient to produce a remission of the disease and the patient needed an allogenic hematopoietic stem cell transplantation (HSCT). In this clinical case, ponatinib was well tolerated and, combined with chemotherapy, was a bridge to HSCT. After HSCT, ponatinib eliminated the presence of the Bcr-FGFR1 fusion protein, which reappeared some months after the transplant. Unfortunately, the fusion protein rose again after the interruption of ponatinib. In conclusion, the potential benefits of ponatinib in this rare and lethal cancer need to be evaluated in every clinical case, also considering the adverse events that have emerged in the setting of CML.

## 3. Solid Tumours

### 3.1. Lung Cancers

#### 3.1.1. Non-Small-Cell Lung Cancer

Ren et al. reported that about 50% specimens from patients affected by non-small-cell lung cancer (NSCLC) overexpress FGFR1. Ponatinib shows potent antiproliferative activity on NSCLC cells expressing high FGFR1 levels and acts through the inhibition of this kinase and its downstream pathways [39].

It is interesting to note that ponatinib shows antiproliferative activity on Ba/F3 cells expressing activated FGFR1-4, with IC_50_ values lower than 40 nM, while it is almost inactive on parental Ba/F3 cells. More importantly, the compound inhibits the growth of 14 cell lines of different tumour types, including lung, endometrial, bladder, gastric, breast and colon cancer with GI_50_ values ranging from of 7 to 181 nM. All these tumours are characterized by FGFR dysregulation. Ponatinib is also active in vivo models of such cancers, where it reduces tumour growth [40].

Dziadziuszko et al. identified the KIT mutation D816G in a tumour sample from a patient with ROS1 positive NSCLC [41].

KIT is a transmembrane TK inhibited by ponatinib. The X-ray structure of ponatinib bound to KIT (PDB ID: 4U0I) indicates that the compound binds to the DFG-out conformation extending into the back pocket of the catalytic site. So, as expected, ponatinib is a type II inhibitor also for KIT [42]. This enzyme, especially in its mutated variants, has been involved in many cancers.

The KIT mutation D816G leads to the constitutively activation of KIT and causes resistance to the ROS1 inhibitor crizotinib in NSCLC patients. Ponatinib inhibits KIT phosphorylation in 293T cells expressing KITD816G. The drug can overcome KIT-mediated resistance in vitro but it remains unknown if the drug can be active on this or other KIT activation mutations also in patients [41].

RET is a receptor TK hyperactivated or mutated in medullary thyroid cancer (MTC) [11,43] and present in mutated or fused forms in some cases of lung and colon cancers [44]. In particular, regarding lung cancer, Nelson-Taylor et al. demonstrated that ponatinib has potent antiproliferative activity in RET fusion-positive LC-2/ad cells of NSCLC and inhibits phosphorylation of the RET fusion proteins, accompanied by a decreased phosphorylation of AKT and ERK1/2. The authors also constructed ponatinib resistant cell lines that maintain activation of the MAPK pathway and suggested that the resistance to ponatinib is probably due to the activity of Ras/MAPK signalling, which leads to the reactivation of oncogenic NRAS and upregulation of epidermal growth factor receptor (EGFR) signalling. This study demonstrated the importance of the development of suitable combination therapy than can avoid or delay the insurgence of resistance [45].

The mutation KIF5B-RET is the most relevant in lung adenocarcinoma, a subtype of NSCLC. Huang et al. established inducible KIF5B-RET transgenic mice and KIF5B-RET-dependent cell lines for preclinical studies on KIF5B-RET-associated lung adenocarcinoma. Ponatinib resulted as the most potent inhibitor among cabozantinib, lenvatinib and vandetanib against KIF5B-RET, both in vitro and in vivo. Importantly, it reduces the volume of xenografted tumours in animals [46]. More recently, Plenker et al. also reported that ponatinib is active on RET-rearranged lung adenocarcinoma models. In particular, the authors identified a novel RET-mutated fusion protein, CCDC6-RET I788N, that confers resistance to the majority of kinase inhibitors, while is sensible to ponatinib [47].

#### 3.1.2. Small-Cell Lung Cancer

Dabir et al. extracted DNA from SCLC (small cell lung cancer) specimens, analysed them by SEQUENOM platform technology and identified the activating M918T RET somatic mutation in a metastatic SCLC tumour sample. Moreover, they found that SCLC cells demonstrated a significantly higher RET expression compared with adenocarcinoma lung cells. Their data suggest that a subpopulation of SCLC patients can benefit from TK inhibitors targeting RET, such as ponatinib, which is active also on the M918T mutation [48].

#### 3.1.3. Breast Cancer Lung Metastases

Shao et al. reported that breast cancer lung metastasis (BCLM)-associated gene expression and lung metastases in breast cancer patients are correlated with c-Jun [49], which is a protein that etherodimerizes with c-Fos to form the AP-1 transcription factor [50]. The authors performed an extensive high throughput screening and identified ponatinib as a potent inhibitor of BCLM in different cellular and mouse models. In particular, ponatinib reduces the expression of BCLM-associated genes by inhibiting c-Jun [49].

### 3.2. Neuroblastoma

Neuroblastoma (NB) is the most common extracranial tumour in infancy, still lacking effective and specific pharmacologic treatments. Many of the kinases targeted by ponatinib, including FGFR1-4, PDGFR but also RET, KIT, FLT3 and VEGFR1 have been shown to be involved in this disease.

Whittle et al. tested ponatinib on different NB cell lines (CHP-134, CHP-212, NGP, LAN-5, SH-EP, SK-N-AS, SK-N-BE and SK-N-SH), on which it shows IC_50_ values in the range 0.9–9.1 µM and decreases cell viability and migration [51]. In an orthotopic xenograft NB model in mice the compound reduces tumour growth and vascularization of the tumour mass. However, although the authors showed that ponatinib has antiangiogenic effect in NB and inhibits different TKs, they did not identify its precise mechanism of action.

Successively, two other groups reported similar results on ponatinib activity against NB both in vitro and in vivo. Li et al. confirmed that ponatinib inhibits proliferation and induces apoptosis in NB cells in a dose dependent manner [52]. One of the mechanism of action, identified by the authors, is the inhibition of FGFR1 and its downstream pathways, which are activated in NB. Ponatinib also increases the cytotoxic effects of doxorubicin in NB cells and inhibits tumour growth in an orthotopic xenograft model in mice. Very recently, Sidarovich et al. screened a library of 349 anticancer agents, which were tested on the three NB cell lines CHP-134, IMR-32 and SK-N-BE. Ponatinib resulted the most promising compound, since it consistently inhibits not only the growth but also migration and invasion of NB cells and, also in this study, is active in inhibiting tumour growth in an orthotopic NB xenograft model in mice [53]. The authors underline that, as suggested by other studies [54] ponatinib could be used at reduced doses (15 mg/day instead of the usual dosage of 45 mg/day), still maintaining its therapeutic activity while decreasing the vascular adverse side effects.

These studies provide the rationale for other preclinical tests of the compound in NB and eventually for clinical studies in patients without alternative treatments.

### 3.3. Glioblastoma

Glioblastoma (GB) is the most common malignancy of the central nervous system. It has been demonstrated that EGFR is hyperactivated and involved in glioma genesis. Ponatinib, at a concentration of 20 nM, has antiproliferative effects, reduces viability, induces apoptosis, inhibits migration and invasion of the U87MG GB cell line. Consistently, it reduces tumour volume in a xenograft model in mice. These results indicate a potential use of ponatinib for GB treatment, even if its side effects of vascular occlusion must be taken in consideration [55].

Another problem regarding the use of ponatinib for GB treatment has been recently pointed out. In fact, a study demonstrated that there is a difference of ponatinib distribution between heterotopic (in mouse flank) and orthotopic (intracranial) models of GB. The drug is effective in the former model, while it is less active in the latter, because of the low total drug concentration reached in the brain and in the brain-around-tumour area [56].

### 3.4. Medullary Thyroid Cancer and Other Endocrine Neoplasias

As already reported, medullary thyroid cancer (MTC) is characterized by RET hyperactivation, due to the presence of enzymatic mutated forms. In particular, notable substitutions regard the valine in position 804, which is the gatekeeper residue, with methionine or leucine. Importantly, the V804 mutations are quite common, both in familial and sporadic cases of MTC, particularly in specific geographic areas [57]. These mutations confer resistance to TK inhibitors such as vandetanib, motesanib and cabozantinib. De Falco et al. reported that ponatinib inhibits RET (WT) and its mutant V804M with IC_50_ values of 25.8 and 33.9 nM, respectively. In cell assays ponatinib has antiproliferative effects on human TT, MZ-CRC-1 and TPC-1 thyroid carcinoma cells that bear endogenous RET alleles and on NIH3T3 fibroblasts transfected with RET mutants. The compounds is active in xenograft models of tumours derived from TT cells, which are medullary thyroid cancer cells harbouring the RETC634W mutation. In the tumour cells a reduction of mitotic index and of RET phosphorylation and signalling have been observed. However, since ponatinib is a multitargeted inhibitor, the authors underlined that it is possible that its action on other kinases, in particular VEGFR-2, may contribute to the activity on MTC cells [58].

Also Mologni et al. reported that ponatinib reduces the growth of RET-expressing cells, while it does not affect RET-negative cells. It potently inhibits both WT and mutant RET kinase, including the drug-resistant V804M/L mutants. Moreover, the authors confirmed the activity of ponatinib against RET in different cell models, by using HEK293 stable clones expressing four disease-associated oncogenic mutants of RET, namely RETC634R (frequently found in multiple endocrine neoplasia 2A), RETM918T (associated with multiple endocrine neoplasia 2B), RETV804L and RETV804M (isolated in familial medullary thyroid carcinoma patients). The four transgenes are significantly inhibited by ponatinib at nanomolar concentrations, although with varying efficiency. This study indicates that ponatinib is a potent pan-RET inhibitor and as in the case of Bcr-Abl, it is probably a type II inhibitor [59].

### 3.5. Gastrointestinal Stromal Tumours

Garner et al. evaluated ponatinib antiproliferative activity on different cell lines harbouring KIT mutants, that are frequently present in gastrointestinal stromal tumours (GIST) [60]. Ponatinib inhibits the growth of cell expressing WT KIT, as well as KIT mutations within exon 11 (V559D and V560G), at the gatekeeper residue (T670I) and within the A-loop (D816H, D820E and A829P), while it is less potent against the mutant V654A. Ponatinib is an order of magnitude more active on A-loop mutants than imatinib, sunitinib and regorafenib, three other KIT inhibitors already approved by FDA and EMA for GIST treatment.

Interestingly, ponatinib has also been tested on three voluntary GIST patients, previously treated with the approved drugs, and, at a dosage of 30 mg/day, it showed encouraging results in two patients [60].

### 3.6. Endometrial Cancer

A subset of patients affected by endometrial cancer (EC) bears activating mutations of FGFR2 and genetic alterations that disrupt the mTOR pathway. These data indicate that the mTOR pathway may act together with FGFR2 in the development of EC [61]. The combination of ponatinib with the mTOR inhibitor ridaforolimus has a synergistic effect on the in vitro growth of EC cell lines bearing an activating FGFR2 mutation and induces tumour regression in an EC xenograft model. This study indicates a potential use of ponatinib in combination with other anticancer agents for the treatment of this tumour.

Regarding the mutant forms of FGFR2 associated whit EC, Sim et al. found that ponatinib is active on five mutants of FGFRs (V561M-FGFR1, N549H-FGFR2, K650E-FGFR3, G697C-FGFR3, N535K-FGFR4), with the strongest inhibitory activity on N549H-FGFR2 (IC_50_ of 0.5 nM) [62]. The compound is slightly less active on the other mutants, with IC_50_ values in the range 1.81–215 nM. Then, the authors demonstrated that ponatinib has a potent antiproliferative and apoptotic effects on the EC cell lines MFE-296, AN3CA, MFE-280 harbouring the activating FGFR2 mutations N549K, K310R/N549K, S252W, respectively. The compound exerts its effects also through the inhibition of ERK, AKT, PLCg/PKC and STAT5 pathways. Moreover ponatinib suppresses the migration and invasion of MFE-296 and AN3CA cells of EC.

### 3.7. Small Cell Carcinoma of the Ovary Hypercalcaemic Type

Ponatinib possesses notable antitumor activity in small cell carcinoma of the ovary hypercalcaemic type (SCCOHT), a rare but aggressive ovarian cancer found in young women. SCCOHT cells express increased levels of PDGFRα and FGFR1, in addition to other receptor TKs such as CSF1R, EPH5A, NTRK2 and ERBB3 [63]. Ponatinib inhibits the proliferation of these cells by targeting multiple kinases. Moreover, the compound is effective in vivo and inhibits the growth of a xenograft model of SCCOHT in mice.

### 3.8. Hepatocellular Carcinoma

FGFR4 is the predominant FGFR isoform of FGFRs in human hepatocytes [64]. FGF19 specifically binds FGFR4 and high FGF19 expression or hyperactivation of FGF19/FGFR4 signalling in hepatocellular carcinoma (HCC) cells is responsible for the resistance to sorafenib, the only TK inhibitor approved for the treatment of this diseases [65]. Ponatinib is able to overcome this resistance by blocking the FGF19/FGFR4 axis in the HCC cell lines MHCC97L, MHCC97H, HepG2 and SMMC7721. This study indicated that the association of ponatinib and sorafenib could be an effective therapeutic approach for HCC treatment.

### 3.9. Rhabdomyosarcoma

Rhabdomyosarcoma (RMS) is the most common childhood soft tissue sarcoma and derives from striated muscle cells. In some patients, amplification and activating mutations of FGFR4 have been detected. In particular, mutations have been identified in the following four FGFR4 residues: Asn535, Val550 (the gatekeeper residue), Ala554 and Gly576 [66]. Li et al. found that ponatinib is the most potent FGFR4 inhibitor among other TK inhibitors and has antiproliferative effects in the nanomolar range on RMS cell lines [67]. The compound reduces the phosphorylation of FGFR4 and of its downstream target STAT3 and induces apoptosis. Interestingly, RMS cells expressing the activating mutations N535K and V550E have increased sensitivity to ponatinib. More importantly, ponatinib inhibits tumour growth in a RMS mouse model expressing mutated FGFR4.

Regarding the binding mode of ponatinib on FGFR4, previously, Lesca et al. co-crystallized the FGFR4-kinase domain with ponatinib and determined that the compound is a type II inhibitor also for this enzyme [66].

### 3.10. Malignant Rhabdoid Tumours

Malignant rhabdoid tumours (MRTs) are lethal paediatric sarcomas, characterized by a deficiency in the SMARCB1 subunit of the SWI/SNF chromatin-remodelling complex, which is involved in tumour development [68]. Wong et al. demonstrated that both PDGFRα and FGFR1 are activated in MRTs and evaluated the effects of ponatinib, which effectively inhibits these kinases, on a panel constituted by 5 SMARCB1-deficient cell lines and 12 WT sarcoma cell lines [69]. The authors found that the compound is effective on two MRT cell lines (A204 and G402) that are SMARCB1-deficient. Further preclinical studies are required to establish the therapeutic window of ponatinib in this type of tumour.

### 3.11. Liposarcoma

A siRNA screening performed by Kanojia et al. revealed that KIT and PTK2 (protein tyrosine kinase 2) are required for survival of liposarcoma, one of the most common soft tissue tumour [70]. Consequently, the authors tested more than 100 TK inhibitors, already approved or in clinical trials, on 11 liposarcoma cell lines and identified ponatinib as the most potent compound against these cells. Ponatinib arrests the cell cycle and causes apoptosis. Its activity is probably due to KIT inhibition. The antiproliferative effects of ponatinib has also been confirmed by the foci formation and clonogenic growth assays. Moreover ponatinib also inhibits the growth of human liposarcoma tumours in mice. From this study, ponatinib resulted endowed with anti-liposarcoma activity, highlighting its potential therapy for treatment of this deadly tumour.

### 3.12. Infantile Myofibromatosis

Mutations of PDGFRβ have been identified in patients affected by infantile myofibromatosis, which is one of the most common soft tissue tumours in children and is associated with a poor prognosis. The mutations are located in the transmembrane, juxtamembrane and kinase domains of PDGFRβ and activate the receptor also in absence of its ligand. The mutant receptors resulted sensitive to the TK inhibitor imatinib, with the exception of D850V. Ponatinib and dasatinib (another multitargeted TK inhibitor) are able to inhibit also this PDGFRβ mutation. The study identified TK inhibitors, especially ponatinib and dasatinib, for the treatment of severe cases of paediatric myofibromatosis [71].

### 3.13. Neurofibromatosis Type 2

Petrilli and colleagues reported that ponatinib is a potential therapeutic compound for the treatment of neurofibromatosis type 2 (NF2) [72], which is a genetic disorder causing multiple benign tumours in the central and peripheral nervous systems, including vestibular schwannomas. NF2 is caused by mutations in the *NF2* gene encoding the tumour suppressor protein merlin (or schwannomin), which regulates many kinase pathways. Merlin-deficient human Schwann cells display hyperactivation of several kinases, including PDGFRα/β and Src. Ponatinib reduces the viability of these cells by decreasing the phosphorylation of PDGFRα/β but also of AKT, p70S6K, MEK1/2, ERK1/2 and STAT3. Further studies addressing ponatinib alone or in combination as a possible therapy for schwannomas in NF2 mouse models are warranted.

## 4. Other Ponatinib Activities

Carver et al. performed a cell based high-throughput assay for the identification of small molecules able to destabilize the oncoprotein KRAS, which is a guanosine nucleotide binding protein regulating many cellular processes, such as cell growth, mobility, invasion. KRAS mutations have been frequently identified in many malignancies, including the most aggressive forms of pancreatic, colorectal, biliary, lung and ovarian cancers [73]. The authors used HeLa cells bearing the fused EGFP-KRASG12V protein and generated an EGFP-KRASG12V fluorescence reporter system implemented for automated screening. With this methodology they tested 465 clinically relevant compounds. Ponatinib, together with AMG-47 (a Lck inhibitor) were identified as the most interesting compounds that could impact the stability of KRAS, by selectively reducing the levels of EGFP-KRASG12V protein in cells.

MEKK2 (MAP3K2) is a cytoplasmic serine-threonine kinase involved in cancer growth and metastasis formation. Noll et al. used a high throughput MEKK2 intrinsic ATPase enzyme assay to identify MEKK2 inhibitors among a collection of known protein kinase inhibitors and identified ponatinib as the most potent compound on this enzyme, with an IC_50_ of 16 nM [74]. Other interesting MEKK2 inhibitors identified during this screening were AT9283, AZD7762, JNJ-7706621, PP121 and hesperidin that have IC_50_ values in the range 18–60 nM [75].

The discoidin domain receptors (DDRs), DDR1 and DDR2, are receptor TKs that are hyperactivated in different pathologies, including fibrosis, atherosclerosis and cancer [76]. Canning et al. reported that ponatinib inhibits DDR1 and DDR2 with an IC_50_ value of 9 nM [77]. Moreover they obtained the crystal structures of the kinase domain of human DDR1 in complex with ponatinib and imatinib and showed that the inhibitors bind also this kinase in a DFG-out mode, consistently with the results obtained from all the other available crystal structures of ponatinib and imatinib in complex with other protein kinases. Moreover the authors defined the structural features determining the binding of DDR-selective inhibitors, that could be used in the treatment of inflammation, fibrosis and lung cancer.

Canning et al. also demonstrated that ponatinib inhibits the serine-threonine kinase RIPK2 (receptor interacting protein kinase 2), which plays roles in the regulation of immune system [78].

This kinase is inhibited by type II inhibitors but not by type I inhibitors (that target the catalytic site in the active enzymatic form) [79]. To confirm this trend, the authors determined the first crystal structure of RIPK2 bound to ponatinib and identified an allosteric site useful for the development of new type II inhibitors. Ponatinib reduces the phosphorylation of RIPK2 and of its downstream pathways in monocytes and macrophages. Moreover it blocks RIPK2 ubiquitination and induction of inflammatory cytokines, and, as a consequence, the nuclear factor κB signalling that is involved in the inflammation process. The authors concluded that ponatinib and new type II inhibitors could be used to treat disease characterized by RIPK2 activation and inflammation-associated cancers [78].

Recently, new studies on ponatinib bioavailability have been performed [80]. It has been determined that the compound and *N*-desmethyl ponatinib (DMP), which is an active metabolite, have a reduced brain accumulation in a mouse model because they are substrates of the multidrug efflux transporter ABCB1 and ABCG2 (ATP-binding cassette B1 and G2). Consistently, ABCG2 deficiency increases DMP plasma levels by about 3-fold. These findings indicate that combinations of ponatinib and ABCG2/ABCB1 inhibitors could be useful in the treatment of patients with brain (micro)metastases positioned behind an intact blood-brain barrier, or with high expression of these transporters in the neoplastic cells.

## 5. Ponatinib in Clinical Trials

In October 2018, ponatinib is presents in 35 interventional clinical trials and in 6 observational studies [81]. Some of them are still recruiting patients, while four trials are just terminated. The main clinical trials testing ponatinib in disease other than CML or Ph^+^ ALL are reported in Table 2.

### 5.1. Recruiting Trials

Seventeen trials are recruiting participants in order to evaluate ponatinib in different malignancies. Obviously, no results have been provided to date but among these trials it is interesting to highlight two innovative studies, which could point the way to a personalized cancer therapy. The first is a phase I trial (NCT02779283), which will evaluate the feasibility of using a functional laboratory based study. More specifically, this trial will determine if the laboratory test can be used to select personalized TK inhibitor therapy in combination with standard chemotherapy in the treatment of patients with newly diagnosed AML and ALL. The second trial is phase II (NCT01620216), recruiting patients who experienced relapsed AML or ALL after a period of improvement or who did not respond to previous treatments. This study is aimed at evaluating the efficacy of a targeted therapy in such patients. It is based on blood sample test from patients in an in vitro assay and the object is to evaluate the response of individual patients to different TK inhibitors.

This procedure may help to determine which kinase inhibitor has the most activity against cancer cells and which could be combined with standard cytotoxic chemotherapy. Based on the results of the assay, the selected groups of patients would receive one of the following drugs: sorafenib, sunitinib, dasatinib, ponatinib, ruxolitinib or idelalisib. The drug would be administered in combination with standard chemotherapy and given to patients on days 8–28 in the absence of disease progression or unacceptable toxicity. The results could demonstrate the benefit of personalized cancer therapy and its future applications in oncology.

### 5.2. Terminated Trials

Among the clinical trials reported in Table 2, two studies were stopped early and participants are no longer being examined or treated. A negative event related to severe adverse effect characterized this clinical status. As previously disclosed, ponatinib has been shown to inhibit FGFR kinase, whose fusion/mutation has been found in NSCLC and in head and neck cancers [39]. Despite the encouraging preclinical results, the relative trial was terminated due to a serious adverse effect (pancreatitis) in one of the two enrolled patients. In a similar way, NCT01838642 was concluded because of a severe gastric haemorrhage and an unexplained death. However, the recruitment status on ClinicalTrials.gov, concerning medullary thyroid cancer, reports that a new study is going to open soon.

### 5.3. Completed Trials

Trials disclosed in this section are completed but frequently, the results appeared so far are not significant and no statistical analysis have been reported. The following discussion collects the available results. Regarding NCT02478164 on refractory-bevacizumab glioblastoma, a set of 15 patients received ponatinib (45 mg, dose daily for 28 days for cycle). Based on the radiologic response, the overall study showed lack of efficacy due to a progression of the disease and a stable disease in 10 patient and 2 patients, respectively. Serious adverse events were recorded in two patients: intracranial haemorrhage and bullous dermatitis of grade 2 and 3, respectively.

No complete results are available for phase II trial (NCT01874665) but the few reported information indicate a progression of the disease in 25 patients on 45, without considering the withdrawal of patients for adverse event or personal choice and physician decision.

### 5.4. Withdrawn Trials

In Table 2 are reported two different trials stopped early, before enrolling its first patients. A pilot evaluation of ponatinib was set up to valuate tumour response in treatment of FGFR mutation positive recurrent or persistent endometrial carcinoma but the trial was withdrawn. According to information provided from ClinicalTrials.gov it could not obtain an IDE (Investigational Device Exemption) on the FGFR2 assay. Subsequently, a dose-escalation study of ponatinib, with and without combination of 5-Azacytidine, in patients with FLT3-mutated acute myeloid leukemia was retired. No additional information are provided regarding causes.

## 6. Conclusions

Ponatinib is a multitargeted TK inhibitor, particularly active as pan-Bcr-Abl inhibitor and for this activity it has been approved by FDA and EMA for the treatment of Ph^+^ leukemias, especially CML, resistant to prior therapies. The compound possesses a higher toxicity compared with all the other TK inhibitor but it is certainly very active and can be used in patients who do not have any other therapeutic option.

Apart from its activity on Ph^+^ leukemias, ponatinib resulted active in different in vitro and in vivo settings of different tumours, where TKs targeted by it are hyperactivated or overexpressed. For this reason, ponatinib is currently being tested in clinical trials for different tumours. In some pathologies, as in the case of refractory glioblastoma, the obtained data have been disappointing. The complete results of the clinical trials terminated or of those just started will give us an overview on the utility of the compound on cancers other than CML, also considering the benefit/toxicity ratio.

In our opinion, at the moment it is difficult to forecast the real utility of ponatinib in different cancers. Presumably, interesting developments on this molecule can be achieved with the use of associations with other TK inhibitors or cytotoxic anticancer drugs, since these combinations could allow a reduction of the dosage of ponatinib and consequently of its severe toxic effects. In this direction, the two innovative clinical trials just reported will give important information on a possible targeted therapy tailored made for patients, on the basis of their response to different TK inhibitors.

## Figures and Tables

**Table 1:** Haematological and solid tumours on which ponatinib has been tested and targeted kinase(s).

Disease	Type of Tumour	Target
Acute myeloid leukemia (AML)	Haematological tumour	FLT3
T-cell acute lymphoblastic leukemia (T-ALL)	Haematological tumour	ERK and STAT5 (in association with PIM inhibitors)
Chronic eosinophilic leukemia (CEL)	Haematological tumour	FIP1L1-PDGFRα
8p11 Myeloproliferative syndrome	Haematological tumour	FGFR1
Lung cancer	Solid tumour	FGFR1, Ret, DDR
Breast cancer lung metastasis (BCLM)	Solid tumour	c-Jun
Neuroblastoma (NB)	Solid tumour	FGFR1-4, PDGFR, RET, KIT, FLT3, VEGFR1
Glioblastoma (GB)	Solid tumour	EGFR
Medullary thyroid cancer (MTC)	Solid tumour	Ret, VEGFR-2
Gastrointestinal stromal tumours (GIST)	Solid tumour	Kit
Endometrial cancer (EC)	Solid tumour	FGFR2
Small cell carcinoma of the ovary hypercalcaemic type (SCCOHT)	Solid tumour	PDGFRα, FGFR1
Hepatocellular carcinoma (HCC)	Solid tumour	FGFR4
Rhabdomyosarcoma (RMS)	Solid tumour	FGFR4
Malignant rhabdoid tumours (MRTs)	Solid tumour	PDGFRα, FGFR1
Liposarcoma	Solid tumour	Kit
Infantile myofibromatosis	Solid tumour	PDGFRβ
Neurofibromatosis type 2 (NF2)	Solid tumour	PDGFRα/β and Src

**Table 2:** Clinical trials and their status on ponatinib, in disease other than CML.

Disease	Target	Phase	Patients	Clinical Trial Status	Clinical Trial Number
AML—ponatinib in association with cytarabine	FLT3-ITD	1–2	40 ^a^	Recruiting	NCT02428543
Lung cancers (adenocarcinoma, SCLC, NSCLC) in patients preselected using specific biomarkers	RET, FGFR	2–3	110 ^a^	Recruiting	NCT01935336
GIST imatinib resistants	Mutated KIT	2	81 ^a^	Recruiting	NCT03171389
Advanced solid tumours	Activating mutations of FGFR1-4, KIT, RET	2	45 ^a^	Recruiting	NCT02272998
Newly diagnosed ALL and AML ponatinib and other TK inhibitors combined with chemotherapy	Multikinases	1	24 ^a^	Recruiting	NCT02779283
Refractory or relapsed ALL and AML and other myelodysplastic syndromes ponatinib and other TK inhibitors	Multikinases	2	24 ^a^	Recruiting	NCT01620216
Advanced NSCLC	RET WT or with translocations	2	9, (20) ^a^	Active	NCT01813734
Biliary cancers	FGFR2 fusion proteins	2	12, (30) ^a^	Active	NCT02265341
NSCLC, head and neck cancers	FRGFRs	2	3, (1) ^b^	Terminated	NCT01761747
Medullary thyroid cancers	RET	2	3	Terminated	NCT01838642
Refractory-bevacizumab glioblastoma	KIT	2	17, (2) ^b^	Completed	NCT02478164
Metastatic and/or unresectable GIST	Mutated KIT	2	45	Completed	NCT01874665
FLT3-Mutated AML ponatinib alone or in combination with azacytidine	Mutated FLT3	1–2	0	Withdrawn	NCT02829840
Endometrial carcinoma	FGFR	N.A. ^c^	0	Withdrawn	NCT01888562

^a^ Estimated enrolment; ^b^ participants withdrew after enrolling and before receiving ponatinib, N.A.; ^c^ Not Applicable.
